# Cell Junctions in Periodontal Health and Disease: An Insight

**DOI:** 10.1055/s-0043-1775726

**Published:** 2023-12-04

**Authors:** Lakshmi Puzhankara, Anjale Rajagopal, Madhurya N. Kedlaya, Shaswata Karmakar, Namratha Nayak, Shashikiran Shanmugasundaram

**Affiliations:** 1 Department of Periodontology, Manipal College of Dental Sciences, Manipal, Manipal Academy of Higher Education, Manipal, Karnataka, India

**Keywords:** anchoring junctions, desmosomes, gap junctions, hemidesmosomes, tight junctions

## Abstract

Cells are the building blocks of all living organisms. The presence of cell junctions such as tight junctions, gap junctions, and anchoring junctions between cells play a role in cell-to-cell communication in periodontal health and disease. A literature search was done in Scopus, PubMed, and Web of Science to gather information about the effect of cell junctions on periodontal health and disease. The presence of tight junction in the oral cavity helps in cell-to-cell adhesiveness and assists in the barrier function. The gap junctions help in controlling growth and development and in the cell signaling process. The presence of desmosomes and hemidesmosomes as anchoring junctions aid in mechanical strength and tissue integrity. Periodontitis is a biofilm-induced disease leading to the destruction of the supporting structures of the tooth. The structures of the periodontium possess multiple cell junctions that play a significant role in periodontal health and disease as well as periodontal tissue healing. This review article provides an insight into the role of cell junctions in periodontal disease and health, and offers concepts for development of therapeutic strategies through manipulation of cell junctions.

## Introduction

Cells are the basic units of a living organism and comprise fluid matrix and organelles containing biochemical components enfolded in a limiting structure called cell membrane. Ever since the components of cells became known, attempts have been made to determine the mechanism of the organization of cells into cell sheets and organ system to perform specific functions 1 and act as a physical barrier against extraneous harmful agents. It has been established that in multicellular organisms, communication between the cells is critical for morphogenesis, differentiation, and growth. Cell junctions play a major role in these cell–cell and cell–matrix communications. These junctions are composed of biochemically and structurally differentiated regions of the plasma membrane of the cell that allow for specific interactions between adjacent cells and between the cell and its external environment. 2 Apart from intercellular communication, cell junctions facilitate the compartmentalization of intravascular and extravascular components and the preservation of cell polarity. The presence of various cell–cell and cell–extracellular matrix junctions enables epithelial cells to secure tight connections with a sustained polarity. 3 The distinctive intercellular junctions are anchoring junctions (AJs), tight junctions (TJs), and gap junctions (GJs). 4 Abnormalities in the organization of these junctions are reported in several genetic and metabolic diseases in human. 5 In the oral cavity, the stratified squamous epithelium is the outmost layer of the oral mucous membrane that protects against microbial infections and mechanical stress. Highly specific junctional complexes between these cells sustain the integrity of the mucosal barrier. The dynamic properties of cell junctions facilitate their rapid rearrangements in physiological and pathological conditions. 6

Periodontal disease is an immunoinflammatory disease that results in progressive destruction of the supporting structures of the teeth. The cell junctions play a critical role in the pathogenesis of periodontal disease as well as in the healing of the periodontal structures. An in-depth knowledge of the structure, components, and functions of the cell junctions would help reveal their role in the mechanisms involved in the pathogenesis of the disease and management of the condition. Several articles describe the cell junctions and their functions 3 4 5 ; however, there is a dearth of literature describing the role of cell junctions in periodontal disease and periodontal healing. This review provides an insight into the role of cell junctions in periodontal health, disease, and their potential role in periodontal healing and management of periodontal diseases.

## Methods

A literature search was performed in PubMed/MEDLINE, SCOPUS, Web of Science, and Google Scholar for all published articles till January 2023 pertaining to cell junctions, periodontal health, disease, and healing. The following search terms, adapted to the specific database, were used: cell junctions OR intercellular junctions OR cell-matrix junctions AND periodontal disease AND Pathogenesis OR periodontal therapy. Data from all clinical trials, cross-sectional studies, case control, cohort studies, literature, and systematic reviews were included. The references of the included studies were checked for additional records. The Gray literature (Google scholar) was also searched. The cross-reference of all studies was searched to include any relevant data. All the articles that did not refer to the cell junctions in periodontal health, disease, and periodontal healing were excluded.

The results of the searches run on different databases were compiled in the Mendeley reference manager (version 1.19.5) and duplicates were removed. For those articles that fulfilled the eligibility criteria, the full articles were retrieved. Any disagreements were mutually discussed between the two reviewers (L.P. and A.R.) and a consensus was reached and the data on cell junctions in periodontal health, disease, and healing were extracted.

## Results

The search yielded 1,639 articles. After removal of duplicates, 1,239 articles were obtained. Titles and abstracts resulted in elimination of 1,149 articles, and 97 articles were subjected to full-text screening. Seventy-five articles pertaining to the role of cell junctions in periodontal health, disease, and healing have been included in the final review. The data obtained have been categorized into the following topics: cell junctions in periodontium, cell junctions in periodontal disease and inflammatory response, microbial influence on cell junctions of periodontium and resultant periodontal disease, cell junction proteins and healing in periodontal tissues, regulation of cell junction, and cell junction proteins for regulation of periodontal health-potential therapeutic strategies.

### Cell Junctions in Periodontium

**Fig. 1:**
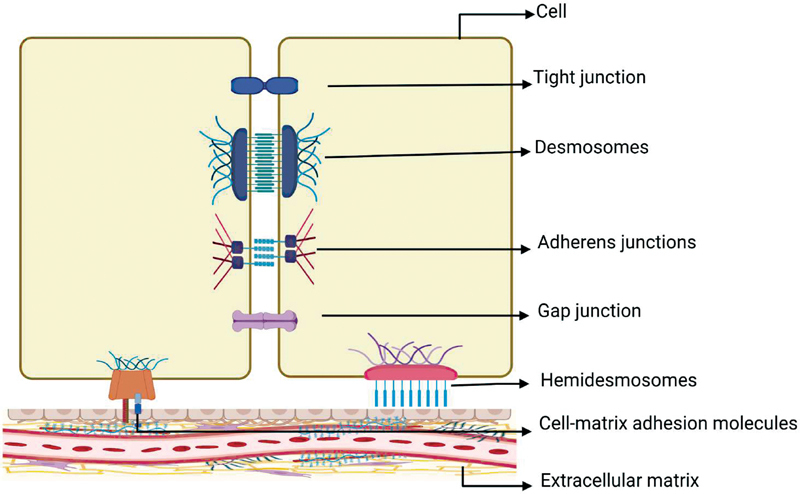
Schematic representation of different cell junctions.

According to their functions, the cell junctions are classified into three categories: TJ or occluding junction, GJ or communicating junction or channel forming, and AJ that consists of adherens junction, desmosome, hemidesmosome, and focal adhesion ( Fig. 1 ).

**Fig. 2:**
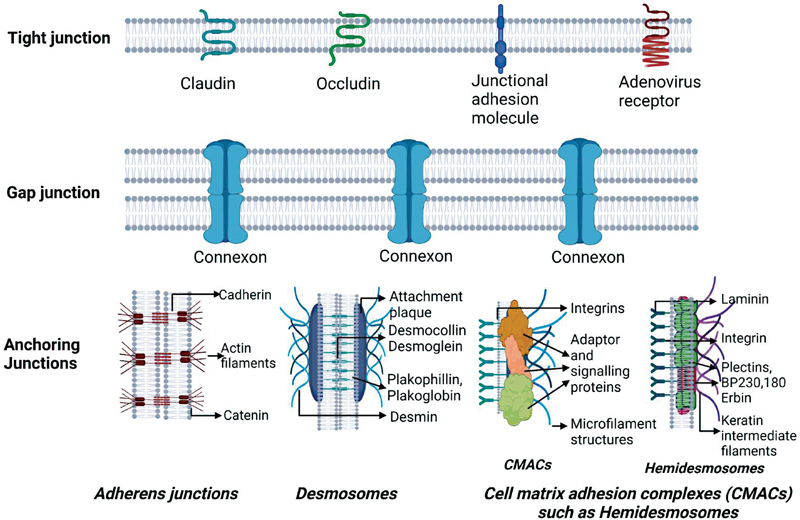
Schematic representation of components of cell junctions.

TJs form a close circumferential connection found between the adjacent cells of the plasma membrane, and their gate function generates an intercellular permeability fence regulating the movement of ions and nonelectrolytes along the paracellular space. The fence function of the TJs facilitates division of the lipid membrane bilayer into basolateral and apical areas, and this facilitates cells to function in a polarized fashion. 7 The GJ helps in controlling the growth and development of an organism, facilitates responses to an external stimulus, regulates homoeostasis, aids in a cell-to-cell adhesion that complements the cadherin- and claudin-mediated bonds, and helps ease direct intercellular communication. 8 The adherens junctions along with the desmosomes form the operational unit of the AJ that aids in enhancing their mechanical strength and integrity, facilitating maintenance of tissue architecture. Four types of AJs exist and include adherens junctions that link cells through actin filament network, desmosomes that utilize intermediate filaments to form a link between cells, hemidesmosomes that link cells to the matrix through intermediate filaments, and other cell–matrix adhesion complexes (CMACs) that link cells to the extracellular matrix (ECM) through actin filaments and regulate cell migration. A schematic representation of the components of the cell junctions are given in figure 2 ( Fig. 2 ). 9

The E-cadherins, which are a component of the adherens junction (AdJ) maintains the structure and integrity in squamous epithelia. The AdJs are abundant in the oral mucosal epithelium and sulcular epithelium, and fewer in the junctional epithelium (JE). 10 Desmosomes are important regulators of health in the oral epithelium. The JE has a lesser number of desmosomes with wide intercellular spaces, 11 which enables an increase in permeability, facilitates gingival crevicular fluid (GCF) flow, permits movement of immune cells, and defense molecules from the connective tissue into the gingival sulcus. 12 Even though limited in number, desmosomal junctions maintain the structural integrity of the JE while preserving the permeability of the structure. Desmosomes and their proteins play a seemingly important role in the development and differentiation of cells in the epithelium. 13 Research shows that these junctions help determine the appropriate cellular phenotypes in tissues as revealed by the regulatory role of the ratio of desmosome isoforms in the stratum corneum and its effect on the degree of corneocyte adhesion and barrier function, 14 and these factors are critical in determining the function of the periodontal structures.

Hemidesmosomes are of paramount importance in oral health as they help attach the JE of the gingiva to the tooth surface. 15 The JE has a standard external basal lamina facing outward to the gingival connective tissue, and a simple and unique internal basal lamina (IBL) that faces inward toward the tooth. This IBL does not contain the common components of a standard basal lamina and anchors the hemidesmosomes that connect the JE onto the tooth. 16 The major transmembrane protein of the hemidesmosome is the α6β4 integrin, which interacts with laminin-332 in the lamina densa. Both α6β4 integrin and laminin-332 play an important role in epithelial cell migration during keratinization and wound healing. 17

Integrin α6β4 is an important hemidesmosomal component that is bound to processed laminin-332 that is present in the IBL of the JE. 18 JE is strategically positioned to act as a wall against bacterial attack through mechanical means and immunological mechanisms but also forms one of the most critical sites for periodontal disease initiation. JE has an external basal lamina or EBL that faces the gingival connective tissue. EBL is composed of lamina lucida (facing the basal keratinocytes) and lamina densa (facing the connective tissue) that forms a true basement membrane. The IBL of the JE is positioned against the enamel 19 to which the basal keratinocytes attach through hemidesmosomes. 20 Basal keratinocytes as well as the epithelial cells of the JE interact with α3 chain of laminin-332 via α3β1 and α6β4 integrins. 21 This binding will support the firm adhesion of basal keratinocytes and maintain the proliferation of cell. 22 α6β4 integrin is co-located with laminin-332, which facilitates the attachment of JE to the tooth. 23 α6β4 integrin binds plectin present in the cell to a complex that collects BP180, which is a transmembrane protein, and BP230, a protein of the plakin family located in the cytoplasm, and these mechanisms allow dermo-epidermal adhesion. 16 β1 integrin-facilitated cell adhesion between the hemidesmosomes facilitates the formation of firm adhesion of JE to tooth structure. 19 These connections maintain the mechanical solidity of hemidesmosomes. 19 The disassembly of hemidesmosomes allows for cell movement during the migration of directly attached to the tooth (DAT) cells. Coronal migration of DAT cells is assumed to begin as a result of phosphorylation of cytoplasmic domain of β4 integrin with a resultant disruption of the bond between β4 integrin and plectin. 24 αvβ6 integrin produced in the JE has the capacity to stimulate latent TGFβ1, which has the function to regulate tissue repair. 19

### Cell Junctions in Periodontal Disease and Inflammatory Response

The development, maturation, and homeostasis of epithelium requires a dynamic synchronization of the cell junctions and the dysfunction of these junctions are correlated with disease conditions including periodontal disease.

Periodontal disease is associated with the release of cytokines of both proinflammatory and anti-inflammatory nature. Proinflammatory cytokines predominantly consist of tumor necrosis factor-α (TNF-α), interleukin-1 (IL-1), IL-4, IL-10, interferon-γ (IFN-γ), and transforming growth factor-β (TGF-β), which can activate the enzymes and transcription factors. More immune cells are recruited to the site with the progression in the inflammatory process. The enzymes secreted by the inflammatory cells and activated by the cytokines degrade the surrounding tissues, and the inflammatory cycle persists with worsening of tissue degradation. 25 The cell junctions appear to affect as well as become affected by the inflammatory cytokines.

A strong correlation exists between the local TNF-α expression, which is a proinflammatory cytokine, and redistribution of TJs, substantiating the fact that the ultrastructure of the TJs was altered by cytokine exposure. 26 A study conducted to test the ability of occludin to form TJ strands in mice fibroblasts showed that occludin is an accessory protein in the TJ strand formation 27 and the expression of Occludin is reduced with an increase in levels of IL-1β mRNA and the microRNA MIR200C-3p seen in inflammation including periodontal inflammation, thereby increasing the permeability of TJ. 28 An increase in the level of serum endotoxin as well as inflammatory cytokines following oral delivery of *Porphyromonas gingivalis* has been shown to result in downregulation of TJ protein-1 zonula occludens -1 (ZO-1) and occludin at the mRNA level. 29

In an inflammatory response, connexin 43 (Cx43), a GJ protein, may be induced in leukocytes, and this will permit the gap junctional communication enabling the extravasation of leukocytes, which are significant components of an inflammatory response. 30 31 Pannexin channels, another component of GJ proteins, include Panx1, Panx2, and Panx3. Of these, Panx3 regulates proliferation and differentiation of keratinocytes, chondrocytes, and osteoblasts. 32 Pannexins have a role on disease progression, and it has been demonstrated that downregulation of pannexin channels may aid in protection against onset of disease and its progression 32 including periodontal disease. Panx1/P2X7 can activate NLRP3 inflammasome and facilitate release of IL-1β *in vitro* , 33 which is involved in inflammatory processes in the oral cavity. Periodontal pathogens can result in a significant decrease in the function of the E-cadherin with alteration in the AJs or associated structures in the oral tissues, which in turn will compromise the barrier function. 34

Periodontal pocket formation, which is a clinical manifestation of periodontal disease, is initiated through a cleavage that occurs within the second or third cell layers of the DAT cell located in the coronal portion of the JE that faces the biofilm. In the process of pocket formation, excessive infiltration of polymorphonuclear neutrophils (PMNs) affects the integrity of the intercellular junctions, detaching the coronal JE from the tooth, 35 which is a pivotal event in the pathogenesis of periodontitis. Subsequent secretion of various chemokines and cytokines with resultant accumulation of neutrophils that release proteases causes disruption and disorganization of intercellular contacts and cell–matrix junctions, and migration of the JE along with ensuing breakdown of the tissue architecture. 36

Functions of the JE, a critical zone of periodontal pocket initiation, is significantly regulated by the cell junctions. Claudins regulate the epithelial barrier at TJs. Although TJs are not a significant component of the JE cell junction, microarray and immunohistochemical analyses have shown that there is a reduction in claudin-1 expression in the JE after chronic lipopolysaccharide (LPS) exposure suggesting that claudin-1 may be involved in epithelial barrier function in the JE even in the absence of TJs. 37 E-cadherins, which are involved in maintaining the function and integrity of adherens and desmosomal epithelial cell junctions, form an important defense mechanism against bacterial invasion in the JE. The levels of E-cadherin were reduced in patients with inflamed gingival tissue. Periodontal pathogens like *P. gingivalis* and *Aggregatibacter actinomycetemcomitans* have been shown to cause a reduction in E-cadherin levels in cultured gingival epithelial cells. 38 Moreover, interruption of E-cadherins in the gastric mucosal epithelium has the ability to increase the permeability of the epithelium, 39 reinforcing the fact that E-cadherins play an important role in epithelial permeability and in defending against bacterial invasion in the JE of the gingiva. Similarly, connexin 26 and connexin 43, which are the GJ proteins, were weaker in a diseased JE. Outer membrane protein 29 (omp-29) from *A. actinomycetemcomitans* and the inflammatory cytokine IL-1β were shown to have the ability to reduce connexin 43 levels and gap junctional intercellular communication (GJIC). 40 These processes may be considered the initial facets of periodontal disease pathogenesis. Kindlin-1, kindlin-2, and kindlin-3 are proteins associated with intracellular integrin activation, and deficiency of kindlin-1 can impair adhesion, migration, and proliferation of cells as integrin activation is deficient in such instances, 40 and it is shown that there is impaired attachment of the JE to the tooth surface in such deficiencies. 41

### Microbial Influence on Cell Junctions of the Periodontium and Resultant Periodontal Disease

*A. actinomycetemcomitans* , *P. gingivalis* , *Tannerella forsythia* , and *Treponema denticola* are periodontal pathogens that have been heavily implicated in the etiology and pathogenesis of periodontal diseases. 6

*P. gingivalis* is keystone pathogen associated with periodontal disease. These pathogens elicit inflammatory responses in the gingival epithelium, along with damage to the epithelial cell layer facilitating colonization of oral soft tissue via penetration of the space between epithelial cells and the connective tissue. By releasing proteolytic known as gingipains, *P. gingivalis* prevents the cell-to-matrix and cell-to-cell adhesions of the keratinocytes. This leads to a negative effect on both the TJs and AJs. 42 *P. gingivalis* ATCC 33277 has been shown to reduce the transepithelial electrical resistance (TEER), which is used to measure the epithelial barrier integrity and function, 38 and gingipain and *P. gingivalis* LPS have the ability to disrupt the AdJ by affecting the cell junction proteins. 43 *P. gingivalis* LPS can reduce the mRNA transcription for occludin and claudin, which in turn leads to disruption of epithelial barrier. 44 *P. gingivalis* fimbriae are virulence factors that facilitate adherence of the pathogen to oral epithelial cells with subsequent invasion into the cells, 44 and degradation of cell adhesion proteins is facilitated by type II fimbriae, 45 indicating that *P. gingivalis* fimbriae have an important role in modifying the epithelial barrier. 46

*A. actinomycetemcomitans* as a whole or its omp-29 can decrease the production of connexin 43, which is a GJ protein in gingival epithelial cells 47 with reduction in GJIC 6 as well as ZO-1 expression. 48 Recombinant cytolethal distending toxin (Cdt), an *A. actinomycetemcomitans* virulence factor, was shown to alter the cytosolic distribution of E-cadherin, which could bring about changes in the scaffolding proteins located intracellularly such as β-catenin and β-actin. 49 These factors lead to remodeling of cell junctions and disruption of the barrier integrity. This causes extensive damage to the gingival tissue. 49

*T. denticola* was found to have the ability to interfere with the cell junctions and result in loose cell contacts and increased permeability with collapsed intercellular spaces 50 along with degradation of the ZO-1 protein. This results in disruption of the epithelial barrier, these deleterious changes may be mediated by dentilisin, a protease present in the outer membrane of *T. denticola* . 46

Metabolic products of anaerobic bacteria such as butyrate have the ability to prevent the proliferation of gingival epithelial cells at low concentrations, whereas at high concentrations, these can induce apoptotic or autophagic death. 51 Sodium butyrate (NaB) disturbs the cell junction protein seen in the AJs such as E-cadherin and TJ protein and claudin-1. 51 *Candida albicans* has been shown to invade the oral epithelium by degrading the E-cadherin by producing a proteinase called Sap5p. This leads to infiltration of the AdJs. 52 It may be inferred from the literature that the pathogens can adversely influence the function of the cell junctions, which facilitates the initiation of the destruction of the supporting structures of the teeth.

The epithelial–mesenchymal transition (EMT) has been linked to the progression of periodontal disease. 53 *P. gingivalis* can produce molecular changes in gingival epithelial cells such as alteration in the glycogen synthase kinase-3 beta (GSK3β) enzyme phosphorylation along with increased expression of transcription factors such as slug and snail with resultant increase in zinc finger E-box-binding homeobox 1 (ZEB1) levels. This results in the inhibition of E-cadherin, upregulation of vimentin, β-catenin, and matrix metalloproteinases (MMPs) 54 with the transition from the epithelial to the mesenchymal phenotype affecting the integrity of the intercellular junctions of the epithelium. Similar findings have been demonstrated by Abdulkareem et al who have shown that exposure of primary oral keratinocytes to *P. gingivalis* and *Fusobacterium nucleatum* can bring about EMT changes. 55

### Cell Junction Proteins and Healing in Periodontal Tissues

The composition of ECM surrounding the keratinocytes is altered during wounding and the cell adhesion receptors on keratinocytes are altered to interact with the ECM. Immediately after wounding, the hemidesmosomal connections to the basement membrane of the keratinocytes at the wound edge are dissolved and the α6β4 integrin distribution around the basal keratinocytes becomes more diffuse 56 along with an increase in the expression of α2β1, α3β1, and α9β1. 57 In the wound keratinocytes, there is also induction of fibronectin receptors, namely, α5β1, αvβ1, and αvβ6 integrins. 58 α9β1 integrin serves as an Extra domain-A (EDA) fibronectin receptor and controls the proliferation of keratinocyte at the wound edge. 59 Cell motility is facilitated by the intermediate adhesiveness to matrix proteins and, hence, integrin–matrix interactions, which are of intermediate strength, are essential for re-epithelialization. This may be achieved via the focalized denaturation of collagen through various mechanisms such as action of MMP-1, which may be induced through an interaction between α2β1 integrin and fibrillar collagens and other factors that facilitate a reduction in the strength of the high-affinity integrin bonding and increased expression of low-affinity integrins. 19 60 αvβ1 integrin, which is a fibronectin receptor with low affinity, facilitates keratinocyte migration as it can support cell attachment without reducing the speed of migration. 61 In addition, when tenascin C binds to fibronectin, the strength of high-affinity α5β1 integrin–fibronectin interface is reduced, and this facilitates cell migration. 62 Other actions such as modulation of TGFβ1-mediated responses by integrins like α3β1 integrin 63 as well as inhibition of β1 integrins, such as α2β1 and α5β1 64 by α3β1 integrin aid in wound healing by reducing the keratinocyte attachment. α6β4 integrin enables epidermal growth factor (EGF) signaling and facilitates wound re-epithelialization. 17

At the completion of re-epithelialization that is marked by the joining and covering of wound surface by migrating cells that originate from wound edges, expression of β1 integrin is downregulated, the binding of α6β4 integrin to laminin-332 is restored, 65 and subsequently hemidesmosomal adhesions will facilitate basement membrane restoration by providing adhesion foci facilitating normal differentiation of keratinocytes. 18 66

In the JE, basal keratinocytes or the DAT cells present at the IBL move along the surface of the enamel. 67 The DAT cells behave like wound keratinocytes with dissolution of hemidesmosomal adhesions and migration toward the gingival sulcus. Laminin-332 is assumed to be secreted by the basal cells of the JE, which permits both migration and firm adhesion of cells based on the processing of laminin-332 molecules. 19 It is an essential component of the epithelium–connective tissue junction and forms a part of the CMACs. The variation in the processing of laminin-332 is related to the expression of three chains designated as α3, β3, and γ2. 68 During laminin processing, the α3 chain may be sliced between the LG3 and LG4 domains by plasmin, which results in transition of laminin-332 to adhesive from migratory. 69 Laminin-332 acts as the main factor facilitating cell migration, 70 and its γ2 chain 71 and β3 chain contribute toward keratinocyte migration. 72 MMP-7 expressed in the JE constitutively and MMPs expressed under inflammatory conditions facilitate laminin-332 processing. 73 The presence of laminin-332 is indispensable for hemidesmosome and basement membrane formation, 74 and it also regulates the formation of granulation tissue in periodontal disease and other inflammatory conditions. 75 Laminin-332 α3 chains can regulate the granulation tissue formation by fibroblasts, 19 75 thereby regulating the healing process in the periodontal tissue in addition to facilitating the epithelial cell migration and healing.

The expression of Cx43, which is a GJ protein, is downregulated in the fibroblasts in the early stages of gingival wound healing, which promote the genes for faster wound healing. 76 Saitoh et al, in their study, concluded that connexin expression alteration affected differentiation and propagation of the keratinocytes in the squamous epithelia. 77 It was also observed that TGF-β1 induced the expression of Cx43 and increased p-Smad2/3 levels. This increased expression of Cx43 further promoted scar tissue formation by the activation of Erk/MMP-1/collagen III pathway. 78

Cx43 has been associated with the extent of differentiation of the precursor cell 79 during odontogenesis as well as repair of the hard tissue components of the periodontium. Cx43 is expressed substantially in human fibroblasts, 80 and this is associated with the capacity of the precursor cells to differentiate into odontoblasts following impairment of primary odontoblasts. 81

Cx43 is found between the adjacent human periodontal ligament (hPDL) cells and these cells have direct cell–cell contact using the cell processes and functional communication through GJs. 82 It has been postulated that the positions of the periodontal ligament (PDL) cells allow them to sense biomechanical stress and communicate with the adjacent bone cells through signals transmitted via the GJs between the PDL cells, facilitating bone remodeling. 82 It has been demonstrated that under conditions of blockage of the GJs or hypoxic stress, RANKL mRNA expression is increased and OPG expression is reduced in the hPDL cells, 82 reinforcing the role of intercellular junctions in periodontal tissue healing and remodeling.

### Regulation of Cell Junction and Cell Junction Proteins for Regulation of Periodontal Health-Potential Therapeutic Strategies

Mechanisms for regulating the inflammatory responses of the periodontal tissue through regulation of cell junction activity may provide potential avenues for periodontal therapy. GJIC, Cx43, and E-cadherin reduction in gingival epithelial cell cultures mediated by *A. actinomycetemcomitans* , omp29, or IL-1β was counteracted by irsogladine maleate through an increase in cyclic antimicrobial peptide (AMP). Irsogladine maleate can also inhibit neutrophil migration induced by *A. actinomycetemcomitans* , thereby regulating the inflammatory responses in periodontal tissue, and reduce the gingival epithelial permeability induced by TNF-α. This action is through prevention of disruption of E-cadherin and claudin-1, thereby regulating epithelial cell barrier. 36 Azithromycin has the ability to reestablish the TEER and E-cadherin expression in human gingival epithelial cells. It also has the ability to suppress the TNF-α-stimulated secretion of IL-8 in human gingival epithelial cells, facilitating an increase in gingival epithelial integrity. 36 The property of inhibition of decrease in E-cadherin expression along with a decrease in *P. gingivalis* LPS penetration was demonstrated by vitamin E and L-ascorbic acid 2-phosphate magnesium salt. 36 Treatment with these antioxidants, which are also known anti-EMT agents, has demonstrated reestablishment of epithelial integrity. 83

#### Beneficial Regulation of Cell Junctions by Oral Bacteria

Beneficial bacteria may act through indirect or direct pathways such as induction of Antimicrobial Peptides (AMPs) through the immune response of the host or demonstrate direct activity against the pathogens that disrupt the epithelial barrier. The bacteria and the bacterial derivatives like 10-hydroxy-cis-12-octadecenoic acid can augment the expression of the TJ-related gene. 46

**Fig. 3:**
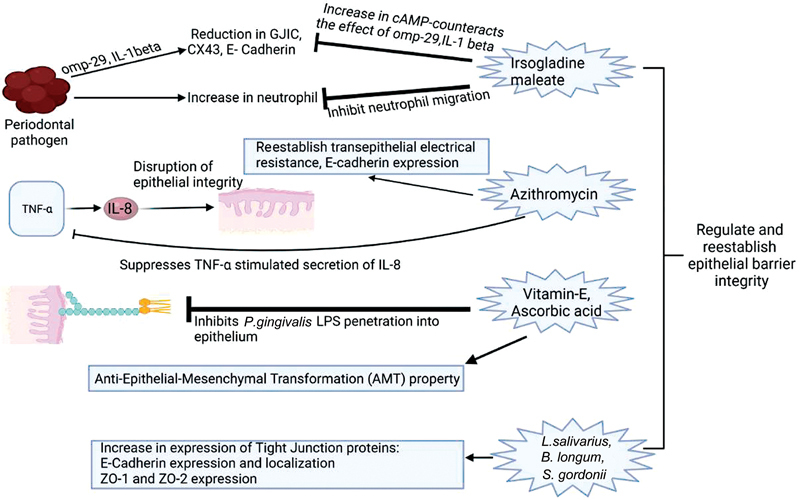
Schematic representation of potential modes of regulation of cell junctions for the management of periodontal disease. cAMP, cyclic adenosine monophosphate; GJIC, gap junctional intercellular communication; IL, interleukin; LPS, lipopolysaccharide; TNF, tumor necrosis factor.

*L. salivarius* can weaken the pathogenic mechanisms that results in disruption of TJs and thereby affect cell–cell adhesions beneficially. There is an increase in E-cadherin localization and E-cadherin expression permitting an upregulation of the epithelial barrier function. 84 Human keratinocyte cells, when treated with *Bifidobacterium longum* ATCC 51870, demonstrated an increase in expression of TJ proteins, increasing the epithelial layer integrity. 46 *Streptococcus gordonii* has the ability to increase ZO-1 and ZO-2 expressions in monolayered oral epithelial cells. 46 A schematic representation of the potential modes of regulation of cell junctions for management of periodontal disease is given in Fig. 3 .

#### Regulation of Cell Junctions through the Influence of Growth Factors

Integrin function, which is an essential element for cell–matrix interactions, is modulated by different growth factors. 85 TGF-β1 upregulates the adhesion of growth factors to fibronectin and the expression of β1 and β3-integrin receptors, 86 and EGF can stimulate β1-integrin production, while platelet-derived growth factor (PDGF) BB increases α5-integrin mRNA levels. 87 In the study by Cáceres et al, 88 it was demonstrated that the adhesion and spreading of fibroblasts to fibronectin matrices was stimulated by platelet-rich plasma (PRP) in a dose-dependent manner and PRP also facilitated the growth of actin stress fibers and paxillin-augmented focal adhesions. 88 Growth factors stimulate the migration of fibroblasts from the connective tissue, which is essential for healing. This occurs through a matrix formed from fibrin and fibronectin, 89 and this matrix provides mechanical support for fibroblasts. 89 Myofibroblastic differentiation is an important step in wound healing. Myofibroblasts facilitate cell-mediated matrix contraction and AdJs have been found to be involved in myofibroblast contractile functions. 91 Myofibroblasts express actin isoform α-Smooth Muscle Actin (a-sma), 90 which is associated with an enhanced capability of cells to contract the cytoskeleton. 91 TGF-β1 induces myofibroblastic phenotype, 90 with a-sma playing a significant role in granulation tissue contraction. 88 Taken together, it may be said that growth factors have the potential to increase wound healing through their action on cell junctions and cell junction components in addition to the other mechanisms of action.

## Conclusion

Cell junctions and cell junction proteins play an important role in maintaining the structural integrity of the oral tissues. They act as a mechanical barrier against the invading pathogens apart from providing protection against microbial attack through molecular mechanisms. Cellular proliferation, differentiation, and migration are also regulated by the cell junctions and cell junction proteins. Aberrations in the cell junctions and cell junction proteins result in pathologies that affect the oral tissues, and hence knowledge of the cell junction proteins and their modulators will help in understanding the pathogenesis of disease processes and also develop therapeutic strategies that target the disease-promoting protein expression or disruption. Therapeutic concepts that target the cell–junction integrity can be utilized to increase the resistance to periodontal disease and facilitate better healing of the periodontal tissues.
